# A personalized computational model of edema formation in myocarditis based on long-axis biventricular MRI images

**DOI:** 10.1186/s12859-019-3139-0

**Published:** 2019-12-10

**Authors:** Ruy Freitas Reis, Juliano Lara Fernandes, Thaiz Ruberti Schmal, Bernardo Martins Rocha, Rodrigo Weber dos Santos, Marcelo Lobosco

**Affiliations:** 1 Department of Computer Science, Universidade Federal de Juiz de Fora, Rua José Lourenço Kelmer, Juiz de Fora, Brazil; 2 Pós Graduação em Modelagem Computacional, Universidade Federal de Juiz de Fora, Rua José Lourenço Kelmer, Juiz de Fora, Brazil; 3 Jose Michel Kalaf Research Institute, Radiologia Clinica de Campinas, Avenida Jose de Souza Campos 840, Campinas, Brazil; 4 Hospital Universitário, Universidade Federal de Juiz de Fora, Rua José Lourenço Kelmer, Juiz de Fora, Brazil

**Keywords:** Computational immunology, Myocarditis, Poroelasticity, Mathematical modeling, Biomechanics

## Abstract

**Background:**

Myocarditis is defined as the inflammation of the myocardium, i.e. the cardiac muscle. Among the reasons that lead to this disease, we may include infections caused by a virus, bacteria, protozoa, fungus, and others. One of the signs of the inflammation is the formation of edema, which may be a consequence of the interaction between interstitial fluid dynamics and immune response. This complex physiological process was mathematically modeled using a nonlinear system of partial differential equations (PDE) based on porous media approach. By combing a model based on Biot’s poroelasticity theory with a model for the immune response we developed a new hydro-mechanical model for inflammatory edema. To verify this new computational model, T2 parametric mapping obtained by Magnetic Resonance (MR) imaging was used to identify the region of edema in a patient diagnosed with unspecific myocarditis.

**Results:**

A patient-specific geometrical model was created using MRI images from the patient with myocarditis. With this model, edema formation was simulated using the proposed hydro-mechanical mathematical model in a two-dimensional domain. The computer simulations allowed us to correlate spatiotemporal dynamics of representative cells of the immune systems, such as leucocytes and the pathogen, with fluid accumulation and cardiac tissue deformation.

**Conclusions:**

This study demonstrates that the proposed mathematical model is a very promising tool to better understand edema formation in myocarditis. Simulations obtained from a patient-specific model reproduced important aspects related to the formation of cardiac edema, its area, position, and shape, and how these features are related to immune response.

## Background

According to the World Health Organization (WHO) and International Society and Federation of Cardiology (ISFC), myocarditis is defined as an inflammatory disease of the heart muscle [1, 2]. Recent studies address myocarditis in postmortem diagnosis of sudden cardiac death ranging from 2% to 44% in young adults [3–5]. Another evidence shows that subclinical myocarditis can be the cause of ventricular fibrillation: 42% of sudden cardiac deaths that occurred among US army recruits were associated with myocarditis [3]. The causes of myocarditis can be divided into infectious, immune-mediated and toxic. This paper focuses on infectious myocarditis which can be bacterial, spirochaetal, fungal, protozoal, parasitic rickettsial and viral [5].

When a living body is affected by an injury or is infected by an antigen, a complex chain of events is triggered, known as inflammatory response. Inflammation is characterized by edema, redness, warmth and pain. All of them emerges from complex physiological processes such as vasodilatation, increase of capillary permeability and pathogen phagocytosis [6–8].

Inflammatory edema is one consequence of an immunological response to an invading pathogen. The immune cells reach the infected area due to an increased vascular permeability, which also allows an increased plasma influx, and fluid accumulation. This process is known as extravasation and is an important mechanism to the edema formation [6].

Edema may be found in several tissues of the body such as lungs [9, 10], hands, arms, legs [11–13] and heart [4, 14, 15]. Heart edema is of great research interest because it is commonly observed in myocarditis and is one of the criteria used in the medical practice to its diagnosis [4, 16]. Although it is invasive and has significant sampling error, endomyocardial biopsy (EMB) is considered the “gold standard” method for detecting myocardial inflammation and interstitial fibrosis. New diagnostic tools have been studied to increase non-invasive diagnostic accuracy of myocarditis and other myocardial pathologies. Cardiovascular magnetic resonance (CMR) imaging provides non-invasive assessment of cardiac function, anatomy and tissue characterization. New techniques have been developed and T2 parametric mapping allows the detection of myocardial edema due to increased water content at the edema regions with greater accuracy [17–19].

Mathematical modeling is a extremely useful tool to uncovers complex physiological dynamics and interactions of biomedical phenomena and systems [20–23]. In particular, distinct approaches have been used to model the interaction between the human immune system (HIS) and a pathogen, such as differential [21, 24, 25] and stochastic [26, 27] equations, cellular automata [28, 29], and agent-based models (ABM) [30–32]. Models based on differential equations are widely used is the field. Ordinary Differential Equations (ODEs) are adopted when only the evolution of HIS populations over time is important, whereas Partial Differential Equations (PDEs) are used to capture spatiotemporal dynamics. Several studies have used ODEs [21, 24, 33, 34] and PDEs [25, 35–39] to model the HIS.

To capture edema formation, the mechanical behavior of tissue and fluids also needs to be considered. Models for Interstitial fluid pressure (IFP) dynamics can be found in the literature [40–42]. One important model that combines IFP dynamics and immune response was first suggested in our previous studies [43, 44]. To include tissue deformation, the classical theory of poroelasticity mechanics of a fluid-saturated porous media that was first proposed in 1941 [45] has been widely used in several studies [46–48]. Two recent works proposed general models in one spatial dimension [49] and a two-dimensions [50] that couple the immune system response with poroelasticity theory. The models were used to describe inflammatory edema formation by also including the modeling of fluid accumulation.

In this work, we extend previous models of edema formation [49, 50] by developing and verifying a patient-specific model for edema formation in myocarditis in a two-dimensional domain. For this purpose, cardiac MRI images were obtained from a patient with myocarditis and were used to develop a specific geometric model of the patient’s heart. In addition, T2 parametric mapping was calculated from MRI images obtained from the same patient to quantitatively describe the edema in terms of location, shape and area. Patient-specific simulations using the new proposed hydro-mechanical mathematical model allowed us to correlate spatiotemporal dynamics of representative cells of the immune system, such as leucocytes and the pathogen, with fluid accumulation and cardiac tissue deformation. Finally, important features of the simulated edema, such as area, location and shape quantitatively matched the clinical ones extracted from the T2 mapping.

## Results

### Patient data

**Fig. 1:**
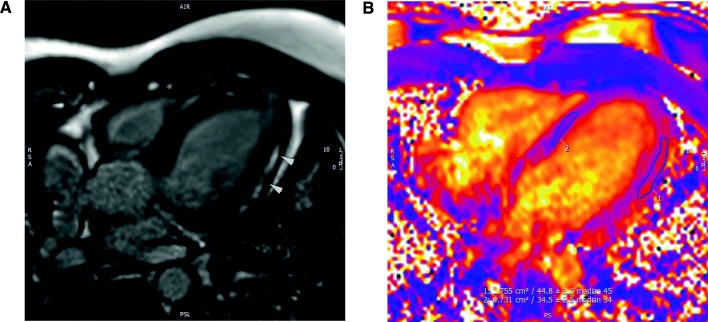
**a** LGE 4-chamber images showing the presence of an area of enhancement in the mid-wall portion of the lateral wall of the left ventricle (arrowheads). Heterogeneous, focal areas of LGE can also be identified in the septal wall. The localization of these areas of LGE are typical for patients with myocarditis and represent one of the main criteria for its diagnostic by MRI. **b** T2 parametric maps of the same patient in A, at the same 4-chamber slice position. The region-of-interest 1 in the lateral wall shows a T2 value of 44.8ms versus a T2 in the septum (ROI 2) of 34.5ms. The increased T2 identified in the lateral wall colocalizes with the LGE area seen in A and suggests the presence of edema/inflammation in comparison to an apparently normal area in the septum

Figure 1 presents Late Gadolinium Enhanced (LGE) image and T2 parametric map obtained in a 4-chamber long axis. LGE image shows the presence of an area of enhancement in the mid-wall portion of the lateral wall of the left ventricle (arrowheads). The localization of these areas of LGE are typical for patients with myocarditis and represent one of the main criteria for its diagnostic by MRI. T2 parametric map at the same 4-chamber slice position quantifies the edema. The region-of-interest 1 (ROI-1) was created using the location indicated by the LGE image. The region-of-interest 2 (ROI-2) was chosen far from this location. In the ROI-1 a T2 value of 44.8ms quantitatively identifies an edema when compared to the T2 value of 34.5ms in ROI-2. Therefore, the increased T2 value identified in the lateral wall colocalized with the LGE area seen in Fig. 1a suggests the presence of edema in comparison to an apparently normal area in the septum (ROI-2). The area of the edema measured by cardiac MRI is equal to 1.755*cm*^2^.

### Numerical results

**Fig. 2:**
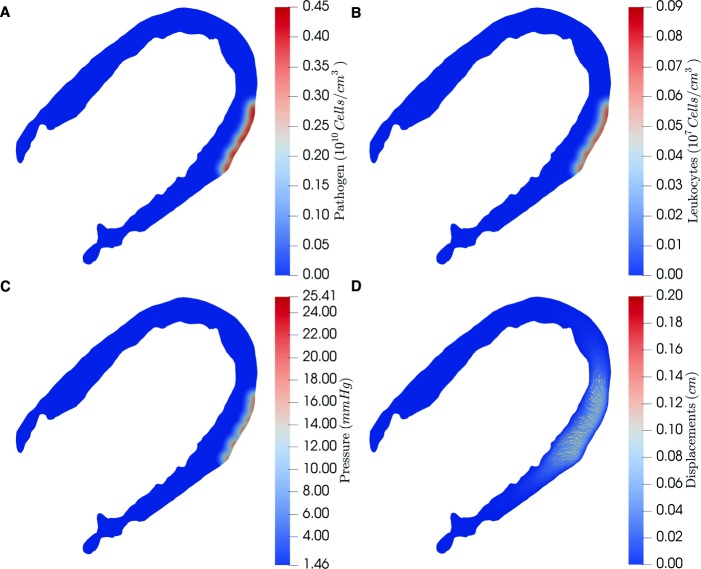
Simulation results of the edema formation using Eqs. (1), (4), (19) and (20) considering the model parameters presented in Tables 1, 2 and 3 with initial and boundary conditions defined in Table 4 after 5h of simulation. Panels A and B show the interactions between a pathogen infection and leukocytes considering an initial infection on edematous epicardium, while panels C and D show its consequences to interstitial fluid pressure and the displacement field

**Fig. 3:**
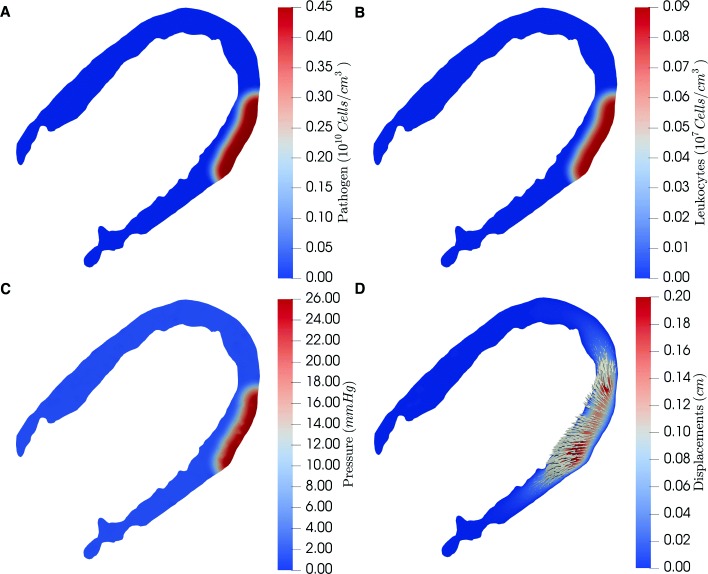
Simulation results of the edema formation using Eqs. (1), (4), (19) and (20) considering the model parameters presented in Tables 1, 2 and 3 with initial and boundary conditions defined in Table 4 after 10h of simulation. Panels **a** and **b** show the interactions between a pathogen infection and leukocytes considering an initial infection on edematous epicardium, while panels **c** and **d** show its consequences to interstitial fluid pressure and the displacement field

Figures 2 and 3 show the simulation result considering Eqs. (1), (4), (19) and (20) after the simulation of 5 and 10 h of infection, respectively. The results were obtained solving the system of PDEs presented in section Methods using as parameters, initial and boundary conditions the values presented in the previous section. Four results are presented: pathogen (panel A) and leukocytes concentration (panel B), pressure (panel C) and displacements (panel D). Although we consider an isotropic elastic tensor, the displacements are larger in the y-axis than in the x-axis (Fig. 3d). This occurs due to the homogeneous Dirichlet boundary conditions imposed on the boundary of the domain (both on the epicardium and on the endocardium). In addition, the magnitudes of the displacements become greater in the border of the infection than in its center. This happens since the points in the vicinity of the infected site are moving in relation to the central points.

**Table 1:** Parameters values for Eq. (1) based on [43, 44, 49, 63]

Name	Value
Porosity (*ϕ*_*f*_)	0.2
Pathogen diffusion coefficient (*D*_*b*_)	$0.00005 \frac {cm^{2}}{h}$
Pathogen reproduction rate (*c*_*p*_)	$0.15 \frac {1}{h}$
Phagocytosis rate (*λ*_*nb*_)	$1.8 \frac {cm^{3}}{h.10^{7}cell}$

**Table 2:** Parameter values for Eq. (4) based on [43, 44, 49, 63]

Name	Value
Porosity (*ϕ*_*f*_)	0.2
Leukocyte diffusion coefficient (*D*_*n*_)	$0.00005 \frac {cm^{2}}{h}$
Chemotaxis rate(*χ*_*nb*_)	$0.0001 \frac {cm^{5}}{h.10^{7}cell}$
Induced apoptose rate(*λ*_*bn*_)	$0.1 \frac {cm^{3}}{h.10^{10}cell}$
Leukocyte capilar permeability (*γ*_*n*_)	$0.1 \frac {cm^{3}}{h.10^{7}cell}$
Leukocyte concentration	
in the blood (*C*_*n, max*_)	0.55×10^7^*cell*
Apoptose rate (*μ*_*n*_)	$0.2 \frac {1}{h}$

**Table 3:** Parameter values for Eqs. (20) and (19) based on [43, 44, 49, 63]

Name	Value
Capillary Pressure (*P*_*c*_)	20.0*mmHg*
Hydraulic permeability (*L*_*p*0_)	3.6x$10^{-8} \frac {cm}{s.mmHg}$
Osmotic reflection coefficient (*σ*_0_)	0.91
Capillary oncotic pressure (*π*_*c*_)	20.0*mmHg*
Interstitial oncotic pressure (*π*_*i*_)	10.0*mmHg*
Pathogen influence in hydraulic permeability (*c*_*bp*_)	$60.0 \frac {cm^{3}}{10^{10}cell}$
Normal lymph flow (*q*_0_)	$0.0001 \frac {cm}{s}$
Lymph flow threshold (*V*_*max*_)	20.0
Increase flow velocity (*K*_*m*_)	6.5*mmHg*
Initial pressure (*P*_0_)	0.0*mmHg*
Exponent (*n*)	5.0
Lamé’s first parameter (*λ*_*s*_)	27.293*kPa*
Shear modulus (*μ*_*s*_)	3.103*kPa*

**Table 4:** Initial and boundary condition

Variable	Initial condition
*C*_*l*_	*C*_*l*_=0∀*x*∈*Ω*
*C*_*p*_	$C_{p} = \left \{\begin {array}{ll}0.2 &\text {on edematous epicardium} \\0 &\text {otherwise}\end {array}\right.$
*P*	*P*=0∀*x*∈*Ω*
Variable	Boundary condition
*C*_*l*_	(*D*_*n*_∇*C*_*l*_−*χ*_*nb*_*C*_*l*_∇*C*_*p*_)·***n***=0∀*x*∈*∂Ω*
*C*_*p*_	*D*_*b*_∇*C*_*p*_·***n***=0∀*x*∈*∂Ω*
*P*	$\frac {K}{\mu }\nabla P\cdot \boldsymbol {n} = 0 \in \partial \Omega $
*U*	*U*=0∀*x*∈*∂Ω*

**Fig. 4:**
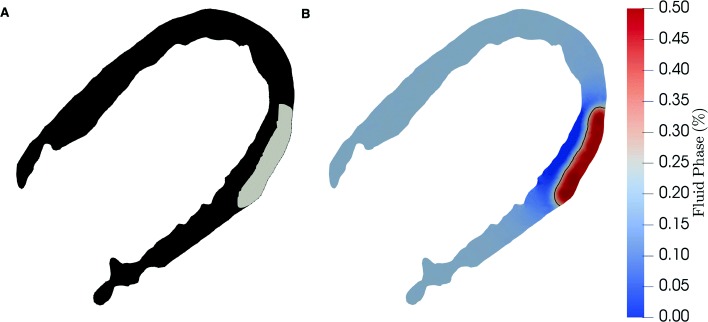
Comparison between the patient edema, identified by a medical doctor, and the results of the numerical simulation. Panel **a** show the T2 mapping imaging exam, Fig. 1b, segmented into a binary image. Panel **b** show the fluid phase distribution after 10 h of simulation and a contour line around the simulated edema

Figure 4a shows the patient edema and Fig. 4b shows the numerical result of the fluid phase obtained by Eq. (7). An isoline is placed in Fig. 4b representing a decrease of 10% in the solid phase. It was used to represent the contour of the edematous tissue in the performed simulation. In addition, the area limited by this isoline and the domain boundary are used to quantify the edematous tissue and compare it to the patient edema area identified by the T2 mapping exam (Fig. 1b). The area of the edema measure by cardiac MRI is equal to 1.755*cm*^2^, whereas the area obtained by the numeric simulation is 1.728*cm*^2^. As one can observe, the relative error is equal to 1.56*%*.

Additional experiments were performed to verify if the random distribution of the lymph vessels affects the final result. Dozens of random distinct distributions of lymph vessels were generated using distinct seeds values, and it was observed that the impacts in the final result were insignificant.

Finally, once *c*_*bp*_ is the parameter which couples the immune system to the hydro-mechanical model, it is important to quantify how the proposed model responds to different values of *c*_*bp*_. So, five simulations were performed varying the parameter *c*_*bp*_, ranging its value from 40.0 to 80.0, and the resulting edematous tissue area was evaluated for each simulation. The results of this analysis is reported in Table 5. As one can observe, a variation of ±20 in *c*_*bp*_ values results in an edema 4.76*%* smaller for *c*_*bp*_=40, and 10,67*%* bigger for *c*_*bp*_=80.

**Table 5:** Influence of distinct *c*_*bp*_ values on the edema area

*c*_*bp*_	Edematous Tissue Area
40	1.646
50	1.709
60	1.729
70	1.815
80	1.913

## Discussions

Figures 3a and b present the numerical results of the simulation of interactions between the human immune system, represented by leukocytes, and pathogens. It is worth to notice that in the simulated scenario it is considered that pathogens start the infection on the epicardium (i.e. outside the domain boundary), more specifically in the boundary of the target edema showed in the T2 mapping exam (Fig. 1b). This infection spreads over time along the simulated myocardium. The simulation starts with no leukocytes in the tissue and after pathogens start to reproduce and spread, leukocytes arrive at the infected tissue from the bloodstream. Moreover, a scenario in which leokocytes and pathogens coexist was imposed to analyze the mechanical deformation of the simulated tissue, but it must be stressed that this immune model is able to reproduce different scenarios [51]. Furthermore, it is important to highlight that the parameter *c*_*bp*_ is the core of the coupling strategy, once it measures the influence of the pathogen to the hydraulic permeability of the microvascular bed wall. So, since the fluid source in the modeled system is the capillary bed, the variation of edema size is directly affected by *c*_*bp*_ as shown in Table 5.

Figures 3c and 3d present the results of the hydro-mechanical model, which simulate the mechanical deformation of the extracellular matrix and tissue cells, both defined as solid phase under the assumptions of a poroelastic media approach. It is important to notice that the fluid accumulation in the infection site is only possible due to the mechanical model, which in this case is triggered by the inflammatory response of the immune system model [49].

Using two types of cardiac MRI, T2 mapping and LGE, the size of the edema was quantitatively measured and highlighted in the MRI T2 mapping (Fig. 1b). Additionally, the imaging exam was segmented into a binary image, presented in Fig. 4a, representing the edematous area in grey and the remaining tissue in black, with the purpose of making easier the comparison between the clinical exam results and the computational ones. Figure 4b shows the fluid phase distribution after 10 h of simulation and a contour line around the simulated edema. The area of the simulated edema matches the area obtained by the T2 mapping exam with an error of 1.56*%*. One can observe that the position and shape of the simulated edema (Fig. 4b) are very similar to the clinical ones (Fig. 4a), which allow us to conclude that the proposed mathematical model is a very promising tool to model the edema formation in myocarditis.

## Conclusions

This study presents a patient-specific model for edema formation in myocarditis. Using patient data obtained with cardiac MRI exams, we were able to create a mesh specific to fit his/her heart. The set of proposed PDEs, that couples the hydro-mechanical model with a mathematical description of the immune system reaction to an invading pathogen, was then solved using this mesh as the domain. The results were able to reproduce the size, position, and shape of myocardial edema. In particular, the edema area obtained by the solution of the numerical method was compared to the edema area obtained clinically.

The major contribution of this study is the quantitative validation of the new hydro-mechanical model using for this purpose clinical data. The obtained result is very promising, indicating that the mathematical model could reproduce some key aspects of edema formation. The quantitative comparison was possible due to the use of the T2 mapping cardiac MRI, a recent technique of cardiac exam which allows quantitative measures of the edematous area. We hope that, in the near future, models like the one presented in this study can be sufficiently precise for reducing the use of animal experiments in early stages of drug testing, as well as to improve drug safety. Additionally, we hope that a better understanding of the processes involved in edema formation in myocarditis can speed up the development of new treatments for patients that are diagnosed with this disease.

The present work could be extended in many distinct ways. The immune system model could be extended to include more details or to model a specific pathogen. In addition, the edema formation in other living tissues could be simulated. The mechanical model presented in this work has some limitations. For example, the influence of the fiber direction in the deformation is not taken into account. Moreover, it is also important the notice that the two-dimensional approach can not consider anisotropic advection-diffusion in the orthogonal direction of the plane axis. To overcome these limitations, in the near future we plan to extend the model to three dimensions using the whole patient-specific heart geometry. Furthermore, in this study we consider that the hydro-mechanical model is coupled with the infection model by means of *L*_*p*_ and *σ* functions, but we do not consider the feedback of the mechanical deformation influencing the infection model, i.e. we consider a one-way coupling strategy. In the near future, we also plan to analyze the influence of the mechanical feedback to the infection model.

## Methods

### Patient data and image processing

Cardiac MRI was performed in a 27-year-old male patient (85*kg*, 171*cm* height) after 5 days of the beginning of symptoms. The patient had undergone a previous invasive angiography that revealed no coronary artery disease. MRI was performed on a 3.0T scanner (Siemens Skyra, Syngo version E11C, Erlangen, Germany) with a 30-channel phased array coil. T2 parametric maps were obtained in a short and 4-chamber long axis using a free breathing sequence with T2-prepared single-shot steady-state free precession (SSFP) imaging [52]. LGE images were obtained after the injection of 0.2*mmol* of gadolinium-based contrast using a sequence with these parameters: gradient-echo inversion recovery (GRE-IR) sequence (TR 6.0*ms*; TE 4.18*ms*; matrix 256×128; FOV 350×260; resolution 1.37×2.0×8*mm*; FA 25o; BW 130*Hz*/*px*; TI 400*ms*; 25 segments; 2 RR intervals; 9 images were obtained in short axis, 3 images in two-chamber view and 3 images in four-chamber views [53]). Figure 1a presents the LGE results.

Post processing was performed using a dedicated workstation with specialized software (CVI42 v5.9, Circle CVI, Calgary, Canada). All images were analyzed by the same reader (JLF, ten years of experience in CMR). For T2 mapping a single region-of-interest was drawn in the septum and lateral wall to obtain the T2 data (Fig. 1b). For LGE images, visual qualitative analysis was performed, classifying the findings as positive or negative for LGE without automated quantification.

### Mathematical model

The mathematical model used to describe the pathophysiology of edema formation was proposed in a previous work [49]. This model includes the inflammatory part which describes the interaction between a pathogen and the human immune system. Along with the inflammatory dynamics, this model also uses a poroelastic model, based on Biot poroelasticity theory, to describe the mechanical deformation of the simulated tissue. Afterwards, this model was proposed to describe the effects of infectious myocarditis in the heart [50].

The model couples the immune response to an extracellular edema formation. The model considers a fluid-saturated porous medium *Ω* to represent the tissue and the pathogen is modelled by:

1 $$ \left\{\begin{array}{ll} \frac{\partial (\phi_{f} C_{p})}{\partial t} = \nabla \cdot (D_{b} \nabla C_{p}) -r_{b} + q_{b} & \ \text{ in}\ \Omega\times I,\\ D_{b}\nabla C_{p} \cdot \boldsymbol{n} = 0 & \ \text{ in}\ \partial \Omega \times I,\\ C_{p}\left(\boldsymbol{x},0\right)=C_{p0}(\boldsymbol{x}) & \ \text{ in}\ \Omega, \end{array}\right.   $$

where $\Omega \subset \mathbb {R}^{2}$ and $I=(0,t_{f}]\subset \mathbb {R}^{+}$ is the time interval, ***n*** is the normal vector outward the boundary domain *∂Ω*, $C_{p}:\Omega \times I\rightarrow \mathbb {R}^{+}$ is the pathogen concentration in interstitial fluid, *ϕ*_*f*_ is the porosity, *D*_*b*_ is the diffusion coefficient of the pathogen through the interstitial fluid, *q*_*b*_ denotes the pathogen reproduction, and *r*_*b*_ denotes the pathogen death due to leukocytes action. Diffusion is defined as the spread of particles from regions of higher concentration to regions of lower concentration. The terms *q*_*b*_ and *r*_*b*_ represent the source of pathogens and phagocytosis of pathogens by leukocytes, respectively, which are defined by:

2 $$\begin{array}{*{20}l}  q_{b} &= c_{p} C_{p}, \end{array} $$

3 $$\begin{array}{*{20}l}  r_{b} &= \lambda_{nb} C_{l} C_{p}, \end{array} $$

where *c*_*p*_ is the pathogen growth rate in the interstitial tissue; *C*_*l*_ is the leukocytes concentration in the interstitial fluid and *λ*_*nb*_ is the leukocytes phagocytosis rate [51].

The leukocyte differential model is represented by:

4 $$ {{}\begin{aligned} \left\{\begin{array}{ll} \frac{\partial (\phi_{f} C_{l})}{\partial t}= \nabla \cdot (D_{n} \nabla C_{l} - \chi_{nb} C_{l} \nabla C_{p}) - r_{n} + q_{n} &\text{ in}\ \Omega\times I,\\ (D_{n}\nabla C_{l} -\chi_{nb} C_{l} \nabla C_{p})\cdot \boldsymbol{n} = 0 & \text{ in}\ \partial \Omega \times I,\\ C_{l}\left(\boldsymbol{x},0\right)=C_{n0}(\boldsymbol{x}) & \text{ in}\ \Omega, \end{array}\right.  \end{aligned}}  $$

where $C_{l}:\Omega \times I\rightarrow \mathbb {R}^{+}$ is the leukocytes concentration in the interstitial fluid, *D*_*n*_ is the diffusion coefficient of the leukocyte through the interstitial tissue, *χ*_*nb*_ is the leukocyte chemotaxis rate, *q*_*n*_ is the leukocyte source, which represents the leukocyte extravasation from bloodstream to interstitium, and *r*_*n*_ denotes the leukocyte death due to both apoptosis and pathogen phagocytosis. These terms are defined by:

5 $$\begin{array}{*{20}l}  q_{n} &= \gamma_{n} C_{p} (C_{n,max} - C_{l}), \end{array} $$

6 $$\begin{array}{*{20}l}  r_{n} &= \lambda_{bn} C_{l} C_{p} + \mu_{n}C_{l}, \end{array} $$

where *C*_*n, max*_ is the leukocytes concentration in the bloodstream, and *γ*_*n*_ is the leukocyte permeability to capillaries microvascular wall [51]. Here, *λ*_*bn*_ represents the death rate after phagocytosis and *μ*_*n*_ represents the natural leukocyte decay, once some of them are preprogrammed to die a short time after they leave the bloodstream [8].

With respect to the mechanical modeling, the stresses related to the tissue can be described by two parts: one which is caused by the hydrostatic pressure of the interstitial fluid filling the pores, and the other caused by the average stress in the solid part, i.e. the extracellular matrix and tissue cells.

Once *Ω* is a fluid-saturated porous medium representing the tissue, therefore a 2-phase system of equations derived from the principle of mass conservation is considered. In this system of equations, one of the equations can be used to describe the fluid (f) and the other the solid (s) phase. The fluid phase is mainly composed of blood plasma, whereas the solid phase represents the extracellular matrix along with the tissue cells. This system is given by:

7 $$ \left\{\begin{array}{ll} \frac{\partial (\phi_{f} \rho_{f})}{\partial t} + \nabla \cdot \left(\phi_{f} \rho_{f} \boldsymbol{v_{f}} \right) = q & \text{for the fluid phase,}\\ \frac{\partial (\phi_{s} \rho_{s})}{\partial t} + \nabla \cdot \left(\phi_{s} \rho_{s} \boldsymbol{v_{s}} \right) = 0 &\text{for the solid phase,} \end{array}\right.   $$

where *q*_*c*_ and *q*_*l*_ represent the capillary bed network and the lymphatic system influence in *Ω*, respectively, and *q*=(*q*_*c*_+*q*_*l*_)*ρ*_*f*_. In addition, *ϕ*_*α*_, *ρ*_*α*_ and ***v***_***α***_ are porosity, density and velocity for each phase, where *α*=*f* for fluid and *α*=*s* for solid.

The plasma flux of the lymphatic capillaries is influenced by the interstitial fluid pressure [54]. The influence of the lymphatic system on the interstitial fluid dynamics, described by the term *q*_*l*_ in Eq. (7), is given by:

8 $$ q_{l}(P)= -\bigg(q_{0}\bigg(1+ \frac{V_{max} (P-P_{0})^{n}}{K_{m}^{n} + (P-P_{0})^{n}}\bigg)\bigg),   $$

where *q*_0_ is the normal lymph flow, *P*_0_ is the normal interstitial fluid pressure. Moreover, *V*_*max*_ is the maximum lymph flow, *K*_*m*_ is the half-live, i.e. the value of *P* which the lymph flow corresponds to $\frac {V_{max}}{2}$, and *n* is the Hill coefficient. These parameters are normally obtained by experimental data [55].

Following our previous work [43], the capillary bed network term *q*_*c*_ is given by:

9 $$ q_{c}(P) = c_{f}(P_{c} - P -\sigma(\pi_{c} - \pi_{i})),   $$

where *c*_*f*_ is the filtering coefficient given by *L*_*p*_(*S*/*V*), *L*_*p*_ is the hydraulic permeability of the microvascular bed wall, and (*S*/*V*) is the vessel surface area per unit volume; *P* and *P*_*c*_ are the fluid pressure in the interstitium and the capillary pressure, respectively; *π*_*c*_ and *π*_*i*_ are the capillary oncotic pressure and the interstitial oncotic pressure due to the plasma protein, respectively; and the reflection coefficient to plasma proteins is denoted by *σ*∈[0,1]. Eq. (9) is known as Starling equation [56] and is widely used [42, 54, 57].

In order to couple the influence of the inflammatory reaction to the fluid phase, the following model [43] for the hydraulic permeability and the oncotic reflection coefficient was used:

10 $$ L_{p}(C_{p}) = L_{p0}(1+c_{bp}C_{p}),   $$

where *L*_*p*0_ is the health tissue hydraulic permeability of the capillary wall, *C*_*p*_ is the pathogen concentration, and *c*_*bp*_ is the influence of the pathogen infection in the hydraulic permeability.

The coupling of oncotic reflection coefficient with the inflammatory response is given by the following relation:

11 $$ \sigma(C_{p}) = \frac{\sigma_{0}}{(1+c_{br}C_{p})},   $$

where *σ*_0_ is the oncotic reflection coefficient in a non-inflamed tissue, *C*_*p*_ is the pathogen concentration, and *c*_*br*_ is the influence of pathogens in the reflection coefficient.

Assuming $\boldsymbol {v_{s}} = \frac {\partial \boldsymbol {U}}{\partial t}$, then Eq. (7) can be rewritten as follows:

12 $$ \left\{\begin{array}{ll} \frac{\partial (\phi_{f} \rho_{f})}{\partial t} + \nabla \cdot \left(\phi_{f} \rho_{f} \boldsymbol{v_{f}} \right) = q & \text{for the fluid phase,}\\ \frac{\partial (\phi_{s} \rho_{s})}{\partial t} + \nabla \cdot \left(\phi_{s} \rho_{s} \frac{\partial \boldsymbol{U}}{\partial t} \right) = 0 &\text{for the solid phase.} \end{array}\right.   $$

Considering that both fluid and solid densities are constant and that *ϕ*_*s*_+*ϕ*_*f*_=1, then Eq. (12) can be rewritten to obtain the following system of equations:

13 $$ \left\{\begin{array}{l} \nabla \cdot \boldsymbol{v_{D}} + \nabla \cdot \frac{\partial \boldsymbol{U}}{\partial t} = q_{c} + q_{l}, \\ \boldsymbol{v_{D}} = -\frac{\boldsymbol{K}}{\mu}\nabla P, \end{array}\right.   $$

where ***v***_***D***_ is the velocity of the fluid phase under a porous media approach, ***K*** is the porous media permeability, and *μ* is the fluid viscosity. The second equation is based on Darcy’s Law, and is often referred to as Darcy velocity [58].

The closure of the PDE system given by Eq. (13) comes when the momentum conservation is considered for each phase, resulting:

14 $$ \left\{\begin{array}{ll} \nabla\cdot(\boldsymbol{\sigma_{s}}) + \boldsymbol{\hat{T_{s}}}= \boldsymbol{0} & \text{for the solid phase,}\\ \nabla\cdot(\boldsymbol{\sigma_{f}}) + \boldsymbol{\hat{T_{f}}}= \boldsymbol{0} & \text{for the fluid phase,} \end{array}\right.  $$

where ***σ***_***f***_ and ***σ***_***s***_ are the fluid and solid phases tensors, respectively, and $\boldsymbol {\hat {T_{f}}}$ and $\boldsymbol {\hat {T_{s}}}$ are the interaction forces between the phases.

Summing up the equations of each phase and considering $\boldsymbol {\hat {T_{f}}} + \boldsymbol {\hat {T_{s}}} = 0$ results in the following equation for the mixture:

15 $$ \nabla \cdot (\boldsymbol{\sigma_{f}} + \boldsymbol{\sigma_{s}}) = \boldsymbol{0}.   $$

The solid phase is considered as an isotropic elastic porous medium, i.e. a Hookean material model [45, 58]. Therefore, the solid phase stress tensor is given by:

16 $$ \boldsymbol{\sigma_{s}} = \lambda_{s} (\nabla \cdot \boldsymbol{U}) \boldsymbol{I} + 2\mu_{s} \boldsymbol{\varepsilon}(\boldsymbol{U}),  $$

where $\varepsilon (\boldsymbol {U}) = \nabla \boldsymbol {U}^{s} = \frac {1}{2}(\nabla \boldsymbol {U} + \nabla \boldsymbol {U}^{T})$, ***I*** is the identity matrix, and *λ*_*s*_ and *μ*_*s*_ are the Lamé parameters. The fluid phase tensor can be described according to the following relation [45, 58]:

17 $$ \boldsymbol{\sigma_{f}} = - P \boldsymbol{I},  $$

where *P* is the hydrostatic pressure at the interstitium and ***I*** is the identity matrix.

Finally, rearranging Eqs. (13) and (15), the following system of equations can be obtained in terms of *P* and ***U***:

18 $$ \left\{\begin{array}{l} (\lambda_{s} + \mu_{s})\nabla (\nabla \cdot \boldsymbol{U}) + \mu_{s} \nabla^{2} \boldsymbol{U} - \nabla P = \mathbf{0},\\ \nabla \cdot \left(\boldsymbol{k} \nabla P\right) = \frac{3}{3\lambda_{s} + 2\mu_{s}} \frac{\partial P}{\partial t} + q, \end{array}\right.  $$

where $\boldsymbol {k} = \frac {\boldsymbol {K}}{\mu }$ is the mobility tensor and ***U*** is the displacement field.

The influence of pressure $P:\Omega \rightarrow \mathbb {R}$ is governed by the following problem:

19 $$ \left\{\begin{array}{ll} \nabla \cdot \left(\boldsymbol{k} \nabla P\right) = \frac{3}{3\lambda_{s} + 2\mu_{s}} \frac{\partial P}{\partial t} + q & \text{ in}\ \Omega \times I, \\ \boldsymbol{k} \nabla P \cdot \boldsymbol{n} = 0 & \text{ on}\ \partial \Omega \times I,\\ P\left(\boldsymbol{x},0\right)=P_{0}(\boldsymbol{x}) & \text{ in}\ \Omega, \end{array}\right.   $$

whereas the governing equation for the displacement field is given by:

20 $$ \left\{\begin{array}{ll} (\lambda_{s} + \mu_{s})\nabla (\nabla \cdot \boldsymbol{U}) + \mu_{s} \nabla^{2} \boldsymbol{U} - \nabla P = \mathbf{0} & \text{ in}\ \Omega,\\ \boldsymbol{U} = \boldsymbol{0} & \text{ on}\ \partial \Omega,\\ \end{array}\right.   $$

where $\boldsymbol {U} : \Omega \rightarrow \mathbb {R}^{2}$; *∂Ω* defines the domain boundary which, in this case, it was applied a homogeneous Dirichlet boundary condition.

### Computational model

To obtain a numerical approximation for all the PDEs presented before we used the finite element method (FEM) due to its flexibility in handling complex geometries and boundaries such as long axis slice of a heart. Here, the FEniCS software library [59, 60] was used to obtain a numerical approximation of the variational formulation of the problems considered in this work. In particular, the models were discretized using linear triangles for spatial discretization and time-derivatives were approximated by a backward difference. The leukocyte model was solved using the SUPG (streamline upwind Petrov-Galerkin) form of the finite element method to ensure stability due to the presence of the convective term [61].

### Mesh generation

**Fig. 5:**
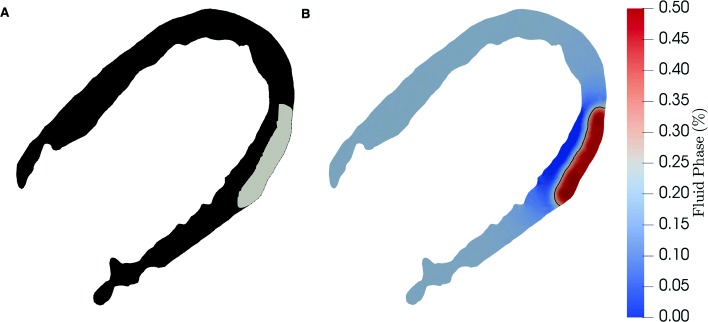
**a** Finite element mesh generated based on Fig. 1b. This mesh represents a slice of the long axis of the left ventricle of the heart of the patient. **b** Distribution of triangular finite elements representing the lymph vessels (white) in the domain. The lymph vessels were uniformly and randomly placed over the domain corresponding to a total of 2.9*%* of the elements

The software Gmsh [62] was used to segment the cardiac MRI exam and generate the finite element mesh which was used to perform the simulations with FEniCS. The geometry description was generated based on Fig. 1b allowing to create a patient-specific 2D finite element mesh with 5015 triangles. Figure 5a shows the resulting mesh. In addition, Gmsh was used to generate a binary image (Fig. 4a), based on the edema area identified by the specialist (Fig. 1b). The idea is to make easier the comparison between the patient edema area and the simulated one (presented in Fig. 4b).

### Simulations setup

Tables 1 and 2 present all parameters values used in Eqs. (1) and (4), respectively. These values were based on previous studies [43, 44, 49, 63]. The values used in Eqs. (19) and (20) are shown in Table 3. To perform the simulations, it was also necessary to set up proper boundary and initial condition of Eqs. (1), (4), (19) and (20). All their functions and values are shown in Table 4.

The patient-specific mesh was created after the segmentation of the long axis cardiac MRI image of the patient (see Methods and Fig. 5a). Lymph vessels represent about 2.9*%* of tissue [64]. To perform the simulations, the finite element mesh was randomly and uniformly filled with lymph vessels, of the size of one element each, with a volume fraction of 2.9*%*. The remaining elements are considered to be only under blood capillary influence. Figure 5b presents an example of a distribution of lymph vessels in the domain, where triangular elements representing lymph vessels are marked in white.

## Data Availability

The data sets supporting the results of this article are included within the article and the references. Model parameters and initial conditions used in simulations are included in this published article. Code to solve the mathematical model can be made available upon request to the authors.

## Abbreviations

CMR: Cardiac magnetic resonance
FEM: Finite element method
HIS: Human immune system
IFP: Interstitial fluid pressure
ISFC: International society and federation of cardiology
MRI: Magnetic resonance imaging
ODE: Ordinary differential equation
PDE: Partial differential equation
SUPG: Streamline upwind Petrov-Galerkin
US: United States
WHO: World Health Organization
